# Concern about passive smoking and tobacco control policies in European countries: An ecological study

**DOI:** 10.1186/1471-2458-12-876

**Published:** 2012-10-15

**Authors:** Marc C Willemsen, Maja Kiselinova, Gera E Nagelhout, Luk Joossens, Ronald A Knibbe

**Affiliations:** 1 STIVORO, Dutch Expert Centre on Tobacco Control, The Hague, The Netherlands; 2 CAPHRI, Maastricht University, Maastricht, The Netherlands; 3 Maastricht University, alumn, Maastricht, The Netherlands; 4 Belgian Foundation against Cancer, Brussels, Belgium; 5 Department of Health Education, Maastricht University, Maastricht, The Netherlands

**Keywords:** Public policy, Smoking, Tobacco control, Passive smoking, Social acceptability, Denormalisation

## Abstract

**Background:**

Because of the magnitude of the global tobacco epidemic, the World Health Organisation developed the Framework Convention on Tobacco Control (FCTC), an international legally binding treaty to control tobacco use. Adoption and implementation of specific tobacco control measures within FCTC is an outcome of a political process, where social norms and public opinion play important roles. The objective of our study was to examine how a country’s level of tobacco control is associated with smoking prevalence, two markers of denormalisation of smoking (social disapproval of smoking and concern about passive smoking), and societal support for tobacco control.

**Methods:**

An ecological study was conducted, using data from two sources. The first source was the Tobacco Control Scale (TCS) from 2011, which quantifies the implementation of tobacco control policies in European Union (EU) countries. Data on smoking prevalence, societal disapproval of smoking, concern about passive smoking, and societal support for policy measures were taken from the Eurobarometer survey of 2009. Data from Eurobarometer surveys were aggregated to country level. Data from the 27 European Union member states were used.

**Results:**

Smoking prevalence rates in 2009 were negatively associated with a country’s TCS 2011 score, although not statistically significant (r = −.25; p = .21). Experience of societal disapproval was positively associated with higher TCS scores, though not significantly (r = .14; p = .48). The same was true for societal support for tobacco control (r = .27; p = .18). The TCS score in 2011 was significantly correlated with concern about passive smoking (r = .42; p =.03). Support for tobacco control measures was also strongly correlated with concern about passive smoking (r = .52, p = .006).

**Conclusions:**

Smokers in countries with a higher TCS score were more concerned about whether their smoke harms others. Further, support for tobacco control measures is higher in countries that have more of these concerned smokers. Concerns about passive smoking seem central in the implementation of tobacco control measures, stressing the importance of continuing to educate the public about the harm from passive smoking.

## Background

Tobacco use continues to cause death, disease, and disability worldwide [1]. Rates of smoking have levelled off or declined in the developed world, while they are increasing in the developing world [2]. Because of the magnitude of the tobacco epidemic, the WHO developed the first legally binding international treaty on public health, the Framework Convention on Tobacco Control (FCTC) [3]. When this treaty went into force in February 2005, governments throughout the world committed to implement a minimum set of tobacco control measures, but were also expected to go beyond that, following additional FCTC Guidelines. Governments now have a wide range of interventions at their disposal to reduce tobacco use, such as adopting high cigarette taxes, banning tobacco advertising and promotion, creating smoke-free public places and worksites, introducing health warnings on cigarette packs, and implementing mass media campaigns to educate about the harm from tobacco. However, more than five years after FCTC came into effect, large differences in implementation levels exist among FCTC ratifying countries [4].

A large and still growing body of literature exists within public health and related disciplines, describing the effectiveness and impact of tobacco control interventions on smoking and health [5-8]. There also is a growing body of literature, predominantly from political sciences, examining how these tobacco control interventions come about in diverse social and political contexts. Adoption and implementation of specific tobacco control interventions is thought to be primarily the outcome of a political process [9-11], where social norms and public opinion may play an important role. Jacobson and Zapawa argue that changes towards more restrictive civil norms regarding smoking facilitate the enactment of legislation, while laws institutionalize nascent civil norms and contribute to a social climate that discourages smoking [12]. Kagan and Nelson, in a discussion of the politics of tobacco regulation in the United States, conclude that, on balance, US tobacco policy reflects shifts in public opinion [13]. A central factor in explaining both the effect of policies on smoking and explaining how these policies originate in society is the concept of denormalisation. Chapman recently argued that “there is a dynamic, synergistic relationship between formal tobacco control interventions and policies, falling smoking prevalence and the increasing range and growth of markers [of denormalisation of tobacco]” [14].

An important aspect besides denormalisation is level of support for policies in the general population. Support for smoke-free legislation is an important determinant of compliance with the legislation [15,16]. Studies found that support for tobacco policies, particularly smoke-free legislation, further increases after implementation [16,17]. However, few studies looked at the association between level of societal support for tobacco control and countries’ tobacco control policy level.

The objective of our study was to examine how a country’s level of tobacco control is associated with markers of denormalisation of smoking, smoking prevalence, and societal support for tobacco control. We conducted an ecological study, using country as the unit of analysis. The European Union provides a good setting for such a study, because tobacco control implementation data are available from the Tobacco Control Scale (TCS) [18], while Eurobarometer surveys [19] provide data on denormalisation, smoking prevalence, and support for tobacco control. A recent study reported on the association between the 2007 TCS score and 2009 Eurobarometer data on smoking prevalence, self-reported exposure to passive smoking, and support for smoking bans [20]. They found that TCS scores were negatively (although not statistically significant) associated with smoking prevalence and positively (and significantly) associated with public support for smoking bans in workplaces. However, they did not examine support for other policies nor markers of denormalisation. We investigated the associations between the more recent 2011 TCS score with Eurobarometer data on societal support for tobacco control measures, smoking prevalence, and markers of denormalisation of smoking.

## Methods

The TCS is based on the six most effective and important tobacco control policies according to the World Bank [18]. A questionnaire was sent to correspondents in all European countries, using a scoring system [18]. This scoring system was developed by a panel of experts who agreed on the allocation of points to the scale and decided on what weight should be given to policies in the scale, reflecting the relative effectiveness of each policy. The maximum score is 100, with subscales for price (maximum 30 points), public place bans (maximum 22 points), public information campaign spending (maximum 15 points), advertising bans (maximum 13 points), health warnings (maximum 10 points), and treatment (10 maximum points). For the present study, the total score was used. Data were taken from the 2011 scale [21].

*Smoking prevalence* data were from the Special Eurobarometer 332, which had fieldwork data conducted in October 2009 [19]. Eurobarometer surveys are periodically conducted by the Gallup Organisation in the 27 countries of the EU, for the European Commission. The Eurobarometer uses a multi-stage random sample design. In each country, a number of sampling points was drawn with probability proportional to population size and population density. In each of the selected sampling points, a starting address was randomly drawn. Further addresses were selected by standard random route procedures, from the initial address. In each household, the respondent was randomly drawn, following the closest birthday rule. Samples sizes varied between 500 (Malta) to 1,550 (Germany), and included all those aged 15 years and older. Interviews are conducted via face-to-face in people’s homes. For all countries, a national weighting procedure was carried that took into account country distributions with respect to gender, age, region and size of locality.

Smoking prevalence was measured in respondents aged 15 years and older with the following question: “Regarding smoking cigarettes, cigars or a pipe, which of the following applies to you?”. Respondents with an affirmative answer to the option “…you smoke at the present time” were regarded to be a smoker.

The Special Eurobarometer 332 includes two items that can be regarded as markers of tobacco denormalisation. The first is *Societal disapproval of smoking,* which is measured with the question: “Has any of the following things led you to think about quitting in the last 12 months …. The society disapproves of smoking?” (Yes, No). This item was also used in other studies as an indicator of the social denormalisation of smoking [22-24]. The other item is *Concern about passive smoking*, measured with the question “Has any of the following things led you to think about quitting in the last 12 months …Concern about the effect of your smoke on non-smokers?” (Yes / No). Both items were measured among smokers who made a quit attempt in the last 12 months.

Data on *societal support for policy measures* were obtained from the Eurobarometer with four questions: “Would you be in favour of or opposed to any of the following measures?” (1) Banning display and advertising of tobacco products in points of sales / shops; (2) Banning the sales of tobacco products via the Internet; (3) Putting picture health warnings on all packages of tobacco products; (4) Increasing taxes on tobacco products. Answering categories on these four questions were: “In favour” and “Opposed”. A scale was formed with a Cronbach’s Alpha of 0.77. These questions were answered by all respondents.

The Central Committee on Research Involving Human Subjects in the Netherlands requires no ethical approval for non-medical survey research.

### Analyses

The relationships between TCS scores, smoking prevalence, the two markers of tobacco denormalisation, and societal support were first examined by means of Pearson’s correlation coefficient and scatter-plots. Next, multiple regression analyses were conduced to examine predictors of TCS 2011 score and predictors of support for tobacco control. We checked for multicollinearity by examining variance inflation factors. For all analyses, SPSS statistical package version 19 was used.

## Results

Smoking prevalence varied between 16% (Sweden) and 42% (Greece), with mean 29.5% (Sd = 5.8). Societal disapproval as a reason for quitting varied between 10% (France, Slovenia) and 42% (Slovakia), with mean 21.3% (Sd 8.4). Concern about passive smoking as a reason to quit varied between 12% (Sweden) and 55% (Ireland), with mean 32.9% (Sd 10.1). Societal support for tobacco control policies was lowest in Austria (51) and highest in Cyprus (80), with mean 62.2 (SD 8.1).

**Figure 1:**
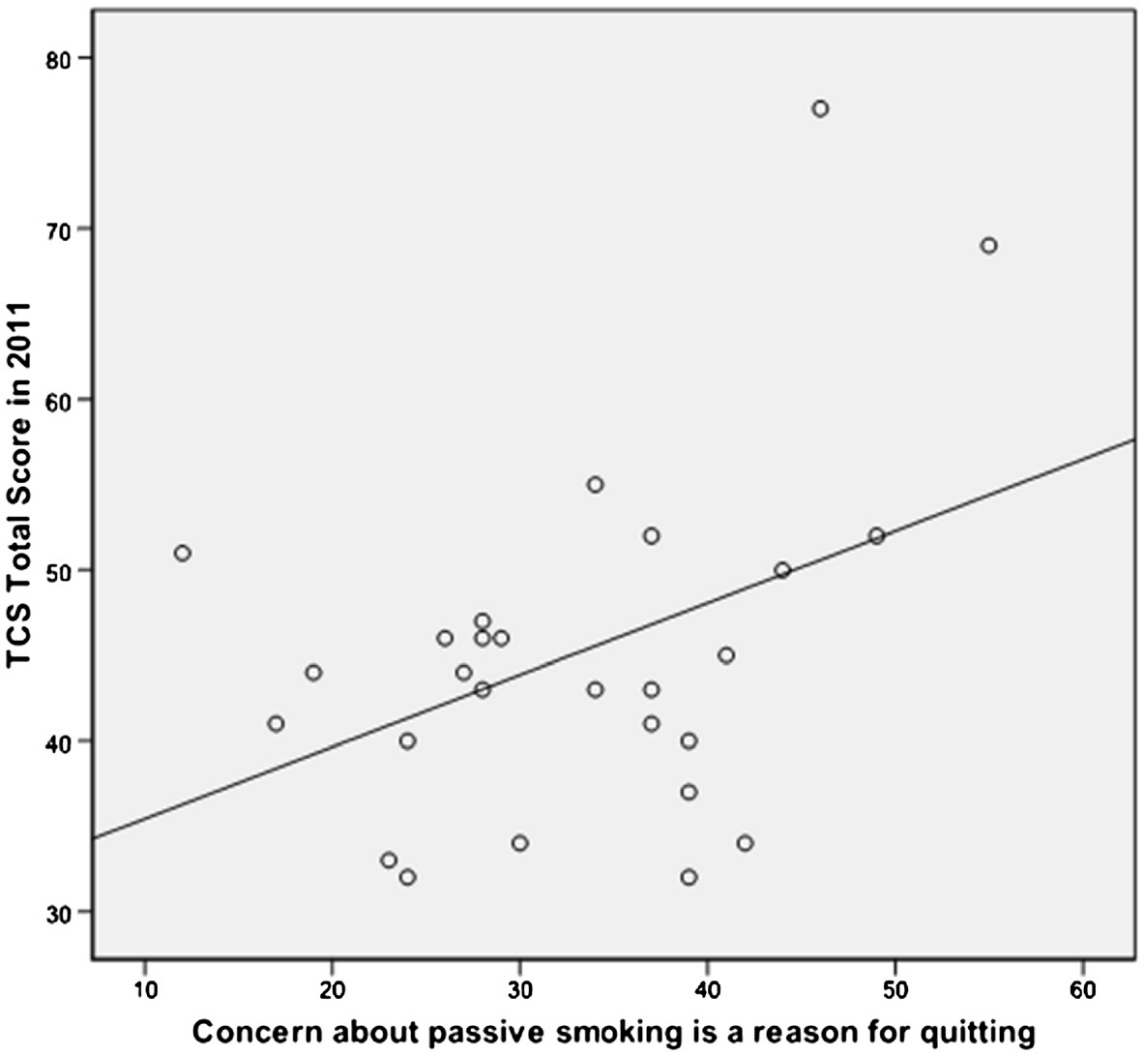
Scatter plot of the association between the total TCS score of 2011 and concern about passive smoking is the reason for quitting.

The correlations between the study variables are presented in Table 1. Smoking prevalence rates in 2009 were negatively associated with a country’s Tobacco Control Scale score in 2011, although not statistically significant (r = −.25; p = .21). The TCS 2011 score was significantly correlated with concern about passive smoking (r = .42; p = .03), meaning that smokers who said that they quit smoking due to concerns about how their smoke affects others, were more likely to live in a country with a more advanced 2011 TCS score. This association is graphically illustrated in Figure 1. This association remained borderline significant in a regression analysis predicting 2011 TCS scores (p = .07), after controlling for social disapproval, societal support, and smoking prevalence (Table 2). Inspection of the scatterplot (Figure 1) reveals that two countries have a strong influence on the total distribution. These are the UK and Ireland, both having a high TCS score and a high proportion of smokers saying that they have quit because of concern about passive smoking. The association is no longer significant without these two countries.

**Table 1:** Correlation matrix (Pearson Correlation Coefficients with 2-tailed p values; N=27)

**Measure**	**% Smokers in 2009**	**TCS 2011**	**Societal Disapproval**	**Concern Passive Smoking**	**Societal Support for TC**
% Smokers in 2009	-				
TCS 2011	-.25 (.21)	-			
Societal disapproval	-.08 (.71)	.14 (.48)	-		
Concern passive smoking	.10 (.61)	.42 (.03)	.47 (.01)	-	
Societal Support for TC	.15 (.45)	.27 (.18)	.31 (.12)	.52 (.01)	-

TC = Tobacco Control; TCS = Tobacco Control Scale.

**Table 2:** Results of multiple linear regression analysis predicting 2011 TCS scores

***Predictors***	***Standardized Beta***	***P***	***VIF***
Societal disapproval	-.13	.54	1.32
Concern about passive smoking	.45	.07	1.61
Societal support for TC	.13	.56	1.40
Proportion smokers in 2009	-.33	.09	1.05

VIF = variance inflation factor.

The data presented in Table 1 further reveal that experience of societal disapproval was positively associated with higher TCS scores, but the correlation was not significant (r = .14; p = .48). The same was true for societal support for tobacco control (r = .27; p = .18). Support for tobacco control measures was strongly correlated with concern about passive smoking (r = .52, p = .006). This remained significant after controlling for proportion of smokers and social disapproval of smoking in a regression analysis (Table 3).

**Table 3:** Results of multiple linear regression analysis predicting societal support for tobacco control

***Predictors***	***Standardized Beta***	***p***	***VIF***
Societal disapproval	.10	.61	1.31
Concern about passive smoking	.46	.03	1.32
Proportion smokers in 2009	.11	.54	1.03

VIF = variance inflation factor.

## Discussion

A previous ecological study found that EU countries with a higher overall TCS score have higher public support for smoke-free legislation (smoking bans) [20]. Our study adds to this with two important findings. First, smokers in EU countries with higher TCS scores are more concerned about the effect of their smoke on others, which can be regarded as a marker of tobacco denormalisation. Second, support for tobacco control measures is higher in countries that have more of these concerned smokers. Together, these findings support the idea that the issue of passive smoking is central to tobacco control in Europe. Our data also suggest that there are still large differences within Europe. The fact that the association between TCS score and concern about passive smoking depends heavily on two countries (the UK and Ireland) which both have a very high TCS score, suggests that these two countries have been much more successful in advancing the implementation of the FCTC measures alongside denormalizing tobacco use, than the rest of Europe.

Despite the paucity of ecological data in the scientific literature, the importance of level of concern about passive smoking was already acknowledged decades ago by the tobacco industry. They initiated social accommodation programs and started covert attempts to deceive the public about the health risks from exposure to tobacco smoke [25], creating controversy about what they called ‘the smoking issue’ [26]. In 1998, a research firm drafted a report, commissioned by Philip Morris, describing and comparing ecological data which they had collected on attitudes towards smoking in a large number of countries across the world [27]. They looked at the relationship between concern about passive smoking and the desire for further restrictions on smoking, finding a strong correlation between the two factors. The authors commented that “this is much stronger than any other driving factor such as social acceptability or disliking the smell”. The industry used these data to tailor their efforts to normalize smoking on a country-to-country basis.

Although the two markers of denormalisation were strongly correlated (r = .47; p = .01), they were not equally strongly associated with the TCS score. The item ‘concern about passive smoking’ showed a high correlation (r = .42; p = .03), but the item measuring ‘social disapproval of smoking’ was only moderately and not significantly associated with TCS scores (r = .14; ns). This suggests that more elaborate measures of tobacco de-normalization are needed. We therefore recommend developing and including well validated, comprehensive measures of tobacco de-normalization in future population surveys.

We found a negative association between a country’s TCS score and the proportion of smokers (r = −.25), albeit not statistically significant (p = .21). This compares well to the results from a previous ecological study using the same number of 27 countries, but comparing the TCS 2007 data with Eurobarometer 2008 data, that found a correlation of -.32 (p = .11) between TCS score and proportion of daily smokers [20]. Both studies probably suffer from the small sample size (N = 27). Another factor obscuring any true association between the two variables might be the low reliability of Eurobarometer surveys. A recent study comparing smoking prevalence proportions from the Eurobarometer 2006 with data from national bureaux of statistics of the 27 EU countries, found large discrepancies, probably due to the inadequate sample sizes in Eurobarometer surveys which hamper an accurate estimation of the proportion of smokers [28].

The study has some limitations. Although the tobacco control score was measured two years after the Eurobarometer scores, it seems not justified to infer strong causality from our ecological data. Although this seems less likely, we cannot rule out the possibility that adoption of more stringent tobacco control policies leads to people becoming more aware and concerned about the harmfulness of smoking. Longitudinal data with representative samples of the population are needed to really disentangle direction of causality and such studies are very much needed. Nevertheless, the fact that the Eurobarometer data preceded the tobacco control score provides some reassurance that concern about passive smoking does indeed drive the implementation of more stringent tobacco control measures. Finally, we cannot rule out the possibility that the association between TCS score and level of concern about passive smoking reflects common underlying factors which have not been controlled for, such as political and cultural differences.

## Conclusion

We used ecological data to examine tobacco denormalisation, an important emerging topic in the field of tobacco control. Our assumption was that countries that have higher levels of tobacco denormalisation would have more advanced tobacco control policies. Our findings suggest that this might be true for one marker of denormalisation, i.e. level of concern about the harm from passive smoking. This also suggests that it remains vital to educate the public about the harm from passive smoking. Even in some developed countries, the awareness of the risks from passive smoking can still be alarmingly low. For example, a recent study found that in the Netherlands only 61% of smokers agree that cigarette smoke is dangerous to non-smokers, whereas this was 83% in the UK, and 96% in France [29].

## Competing interests

The authors declare that they have no competing interests.

## Authors' contributions

MW conceived and designed the study, coordinated and carried out statistical analyses and drafted the manuscript. MK participated in the design of the study, carried out the data collection, conducted statistical analyses and helped draft the manuscript. RK participated in the design of the study and co-supervised the study. LJ and GN helped draft the manuscript. All authors contributed to the writing of the manuscript and critically reviewed the final version. All authors read and approved the final manuscript.

## Pre-publication history

The pre-publication history for this paper can be accessed here:

http://www.biomedcentral.com/1471-2458/12/876/prepub
